# Integrative modeling of the cell: Cell modeling

**DOI:** 10.3724/abbs.2022115

**Published:** 2022-08-25

**Authors:** Xianni Zhong, Jihui Zhao, Liping Sun

**Affiliations:** 1 iHuman Institute ShanghaiTech University Shanghai 201210 China; 2 School of Life Science and Technology ShanghaiTech University Shanghai 201210 China

**Keywords:** whole-cell modeling, integrative modeling, compartment model, cell biology

## Abstract

A whole-cell model represents certain aspects of the cell structure and/or function. Due to the high complexity of the cell, an integrative modeling approach is often taken to utilize all available information including experimental data, prior knowledge and prior models. In this review, we summarize an emerging workflow of whole-cell modeling into five steps: (i) gather information; (ii) represent the modeled system into modules; (iii) translate input information into scoring function; (iv) sample the whole-cell model; (v) validate and interpret the model. In particular, we propose the integrative modeling of the cell by combining available (whole-cell) models to maximize the accuracy, precision, and completeness. In addition, we list quantitative predictions of various aspects of cell biology from existing whole-cell models. Moreover, we discuss the remaining challenges and future directions, and highlight the opportunity to establish an integrative spatiotemporal multi-scale whole-cell model based on a community approach.

## Introduction

Cells, as the fundamental unit of life, have been extensively studied by various experimental and computational techniques. For example, the census of components of cells can be detected and quantified from molecular levels (via immune functional assays (IFA) [1], mass spectrometry (MS) [ 2, 3] , *etc*.) to organelle scales (via soft X-ray tomography (SXT) [4], cryo-electron tomography (cryo-ET) [5], *etc*.). Cell signaling pathways can be illustrated by MS-based proteomics and metabolomics [ 6, 7] . However, each experimental technique only sheds light on limited aspects of cell structure or function from a single cell or a subset of cells. Commonly used computational methods often produce structure models of biomolecules ( *e.g.*, homology models of protein structures [8]) or mathematical models of certain cellular pathways ( *e.g.*, GPCR signaling pathways [9]), which are insufficient to learn new biology in the context of the whole cell.

A whole-cell model describes certain aspects of the entire cell as a function of its components and relationships among them [10]. The best way to model the cell is to integrate all available information including experimental data, prior knowledge and prior models to generate an accurate, precise and complete model of the cell. A comprehensive whole-cell model should have the following attributes: (i) a detailed census of the identities and quantities of its components; (ii) the spatial and temporal distribution of these components; (iii) a multi-scale description from atoms to cellular compartments [11]; (iv) hierarchical topology of the cell revealing the functional relationships among components; (v) heterogeneity among individual cells of the same subtype, or among cells of different subtypes [ 12– 15] and among cells from different individuals and/or from different organisms; (vi) integration of input information across varying representations; (vii) proper quantification of its uncertainty [16]; (viii) rationalization of existing experimental observation and prediction of new biology; (ix) possibility to iterate over new information and to employ newly developed methods [10]. Ultimately, these cell models are expected to provide quantitative predictions on various aspects of cell biology (for details, see Whole-cell Model Prediction on Cell Biology).

Extensive efforts have been made for integrative modeling of the cell over recent years, including integration of stochastic dynamics and spatial organization of the cell [ 17– 19] , integration of biochemical reactions in different cellular compartments revealing spatiotemporal modulation of cellular processes [19], integration of mathematical equations recapitulating biochemical pathway across the cell [ 20– 22] , integration of models across varying representations towards a comprehensive model of the cell [ 10, 23, 24] , *etc*. For example, Ghaemi *et al*. [19] revealed the influence of spatial organization on RNA splicing by incorporating complex biochemical networks into a spatially-resolved human cell model (whole-cell compartment model). Recently, Thornburg *et al*. [25] presented a whole-cell fully dynamical kinetic model of JCVi-syn3A to reveal how emergent imbalances lead to slowdowns in the rates of transcription and translation. Karr *et al*. [22] constructed a computational model of a human pathogen including all of its molecular components and their interactions to predict phenotype from genotype based on numerous differential equations (whole-cell mathematical model). Later on, Agmon *et al*. [ 26, 27] proposed a software tool, *Vivarium*, to compose heterogeneous datasets and diverse mechanistic modeling strategies into an integrated multi-scale model. Furthermore, Qin *et al*. [28] built a multi-scale integrated cell model to reveal the hierarchical map of human cell architecture by fusing protein images and interactions (whole-cell structure model). More recently, Raveh *et al*. [24] developed a Bayesian metamodeling approach to construct a comprehensive model of glucose-stimulated insulin secretion pathway by integrating heterogeneous models of complex biological systems ( *e.g.*, coarse-grained spatiotemporal simulation [29], ordinary differential equations (ODEs) [30] and molecular network model [31]).

Additionally, many other modeling platforms have been developed to simulate cellular processes and visually reconstruct cellular landscapes [32]. For example, VCell simulates various molecular mechanisms of a cell [33]; MCELL simulates ligand diffusion and chemical signaling reactions in a cell [34], and E-CELL simulates cell behaviors using differential equations [35]. In addition, CellPAINT illustrates the molecular organization of the cell by a popular digital painting software based on the experimental data from many different laboratories [ 36, 37] . From the above-mentioned examples, we can observe that each of these cell models and platforms provides some degree of insight and represents important milestones in modeling the whole cell.

Here, we summarize a generally applicable integrative whole-cell modeling workflow from input information to the output whole-cell model. This workflow emphasizes a modular representation due to the high complexity of the cell where the output model is either built by constructing and integrating intermediate models for individual modules or by integrating information over modules directly. We highlight a recent substantive development of an integrative modeling approach, namely Bayesian metamodeling, to integrate data and models of various aspects of a cell including whole-cell models towards a more accurate, precise, and complete whole-cell model [24]. Moreover, we review various predictions of new biology from recently developed whole-cell models. Finally, we discuss the remaining challenges, in particular, the need for a multi-disciplinary research community to facilitate the construction of an integrative spatiotemporal multi-scale whole-cell model.

## Whole-cell Modeling Workflow

**Figure 1:**
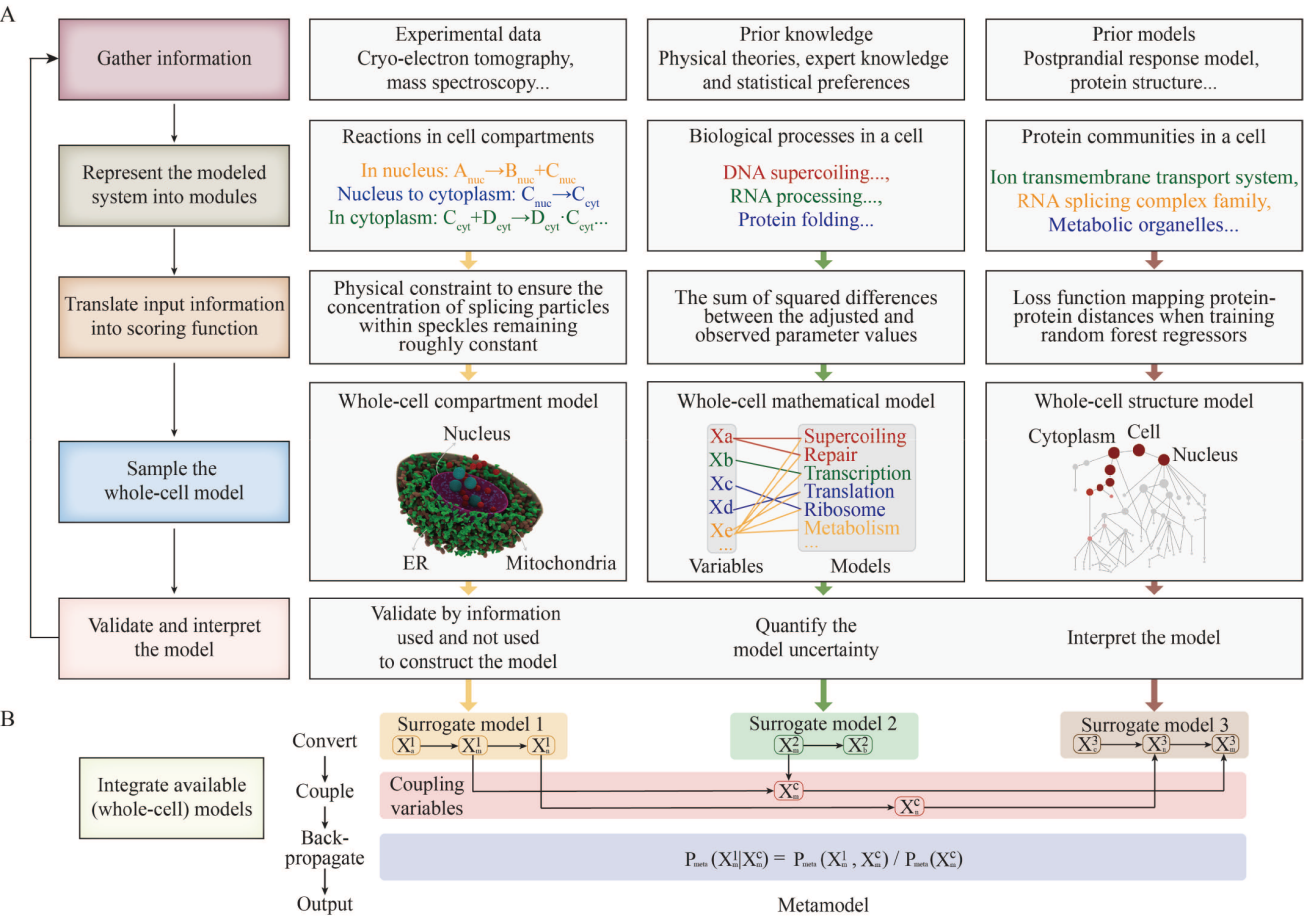
Figure 1 Description of the whole-cell modeling workflow (A) The whole-cell modeling workflow is summarized into five steps using three examples [ 19, 22, 28] . First, gather information such as reactions of components, ODEs and protein distances. Second, represent the modeled system into modules ( e.g., cell compartments, cellular processes and protein communities) based on input information. Third, translate input information into scores, which can be physical restraints (whole-cell compartment model), the sum of square differences (whole-cell mathematical model) and loss functions (whole-cell structure model). Fourth, sample the whole-cell model by integrating intermediate models of modules or information over modules directly, followed by custom optimizations ( e.g., refinement of model parameters and model concordance with previous studies). Fifth, validate the model by information used and not used to construct the model, quantify the model uncertainty, and interpret the model. Iteration of the five steps allows to refine the cell model when there is new information, modeling methods or computational capacity. (B) Integrating available (whole-cell) models will yield a more accurate, precise and complete whole-cell model [24].

An integrative modeling approach is often used to model complex systems such as the whole-cell by combining various types of experimental data, prior knowledge and prior models of different aspects of the cell. The workflow comprises five steps ( Figure 1): (i) gather information; (ii) represent the modeled system into modules; (iii) translate input information into scoring function; (iv) sample the whole-cell model; (v) validate and interpret the model. To the best of our knowledge, existing whole-cell models (summarized in Table 1) are built following a similar workflow. The next paragraphs describe each step in detail using three representative whole-cell models: (i) whole-cell compartment model [19]; (ii) whole-cell mathematical model [22]; and (iii) whole-cell structure model [28]. In addition, we depict a special case of integrative modeling, Bayesian metamodeling, which integrates available (whole-cell) models of various aspects of a cell and its parts across heterogeneous representations.

**Table:** **Table 1** Whole-cell models and their predictions

Model	Prediction	Authors	Year	Reference
E-CELL	Minimal gene complement for a self-replicating cell	Tomita *et al*.	1999	[35]
Whole-cell mathematical model	Cellular phenotypes arising from individual molecules and their interactions of life cycle	Karr *et al*.	2012	[22]
Large-scale mechanistic model of *Escherichia coli*	Protein half-lives for the specific genes	Macklin *et al*.	2020	[20]
Whole-cell compartment model	Effect of the spatial distribution of macromolecules on the RNA splicing	Ghaemi *et al*.	2020	[19]
Minimum stochastic model of the β-cell	Concentration perturbations, disruption of patterning, and their changes to the phase of the signal	Tenner *et al*.	2020	[ 33, 38]
Multi-scale model of chemotactic *E. coli*	Real flux profiles measured in colonies of cells	Agmon *et al*.	2020	[27]
Whole-cell structure model	New protein communities of the modeled system	Qin *et al*.	2021	[28]
Bayesian metamodel	Effect of incretins and other small molecule ligands on systemic insulin response	Raveh *et al*.	2021	[24]
Whole-cell protein model	New particle positions and their velocities at a specific time in a biological process	Das *et al*.	2021	[39]
JCVi-syn3A model	Emergent imbalances leading to slowdowns in the rates of transcription and translation	Thornburg *et al*.	2022	[25]

### Step 1: Gather information

A whole-cell model is built based on multiple types of input information of the cell, including experimental data, prior knowledge and prior models. Note that overlaps might occur among these three types of input information depending on specific modeled systems and modeling approaches. For example, a 3D protein structure determined using X-ray crystallography is often seen as experimental data, but can be used as a prior model in the integrative modeling of protein complexes [40].

Over the past few years, numerous experimental data have been collected and made accessible to the public, which greatly accelerates the development and application of modeling methods [ 41, 42] . There are various experimental data including structural features ranging from molecular to subcellular and cellular scales collected using cryogenic electron microscopy [43], cryo-ET [44], SXT [29], coherent diffraction imaging [45], *etc*.; spatiotemporal patterns of certain proteins or organelles of a cell collected using fluorescence lifetime imaging microscopy [46]; signaling and metabolic pathways in a cell measured by MS-based proteomics and metabolomics [47]; and many others [48]. Databases have been developed to curate different types of data and facilitate a wide range of data interpretation and modeling efforts such as OmicsDI [49] and Datanator [50]. The whole-cell compartment model ( Figure 1, left panel) is constructed using various experimental data, such as proteomics data [51], cryo-electron tomographic data [52], hydroxyl radical probing and mass spectrometric data [53]. The whole-cell structure model ( Figure 1, right panel) is established based on the immunofluorescence images in the Human Protein Atlas [54] and affinity purifications (APs) in BioPlex [55]. Prior knowledge usually refers to statistical preferences, expert knowledge and physical theory. For example, chi-square statistics are often used to compare observed results with expected results [56]. In the whole-cell compartment model ( Figure 1, left panel), the concentration of splicing particles within speckles remains roughly constant according to the physical constraint of phase separation [19].

In addition, numerous prior models have been deposited in publicly accessible databases, including the worldwide Protein Data Bank (wwPDB) [41], the endocrine and neural dynamics section of the national institute of health [57], BioModels [ 58, 59] , BioSimulators [60] and others [61]. As an example, in the whole-cell mathematical model ( Figure 1, middle panel), some ODEs and their parameters are implemented as originally reported or are carefully reconciled [22]. Integration of various types of information maximizes the comprehensiveness of the whole-cell model.

### Step 2: Represent the modeled system into modules

Model representation is determined by the input information. Due to the extensive amount of computing tasks for whole-cell modeling, modular representation is often adopted for different components and/or processes in a cell. Input information is divided into modules either in this step or in step 4 when computing the model.

Representation of a model specifies the variables whose values will be determined by modeling [23]. There are various types of representations at different spatiotemporal scales ( *e.g.*, subcellular structures to cells and milliseconds to hours) and granularities ( *e.g.*, atoms to subcellular structures and femtoseconds to seconds) to depict different aspects of a cell. For example, a whole-cell model can be represented as compartments ( *e.g.*, organelles and microdomains) to concisely describe the structures, dynamics and interactions of its components. Model representation is designed based on the interpretation of input information. It is a critical step, which largely affects choices of the subsequent scoring function and sampling methods ( *e.g.*, different coarse levels of organelles affect the resolution of the scoring function). When the modeled system is as complex as the cell, investigators have often modularized the cell according to its biological components/functions. Consequently, modules are defined mainly according to the representation of the modeled system, which can be different compartments of a cell ( *e.g.*, nucleus and cytoplasm) [ 19, 39] , different cellular processes ( *e.g.*, chromosome segregation, transcription and protein folding) in ODE-, flux balance analysis (FBA)-based mathematical models [22], and different protein communities (subcellular components, *e.g.*, ion transmembrane transport system and RNA splicing complex family) in cell structure models [28].

### Step 3: Translate input information into scoring function

Information can be used to construct a scoring function to quantify the degree of consistency between the model and the input information [23]. Data interpretation is essential for establishing the functional form and parameters of the scoring function. The scoring function is then applied to restrain different aspects of a model such as structures ( *e.g.*, pairwise distances between proteins in a cell [28]), dynamics ( *e.g.*, excluded volumes between cell components [19]) and values of model parameters ( *e.g.*, parameters in ODEs [22]). An acceptable score is the sufficient degree that the model satisfies input information. Several commonly used scoring functions include excluded volumes, physical constraints [ 19, 62] , the sum of square differences [22], loss functions in machine learning models [ 28, 63, 64] , and others [65]. For example, Ghaemi *et al*. [19] scored the whole-cell compartment model by physical constraints ( *e.g.*, excluded volume, size and number of nuclear speckles in a certain range as well as roughly constant concentration of splicing particles within speckles). One example of the acceptable score is that the size of nuclear speckles in the compartment model falls in the range of 1.4 to 1 μm. Karr *et al*. [22] optimized the whole-cell mathematical model according to the sum of square differences between the adjusted and observed parameter values among all sets of parameters. Qin *et al*. [28] used loss functions to map protein-protein distances when training random forest regressors.

### Step 4: Sample the whole-cell model

Ideally, good-scoring models are found by systematic enumeration of the model phase space, going through every possible model with sufficient granularity [ 23, 66] . Due to the high computational cost, other methods are often used to simplify the sampling process ( *e.g.* Monte Carlo sampling [67], Molecular Dynamics [68], Brownian Dynamics [39], Quasi-Newton methods [69] and collocation methods [70]). Sufficient sampling is required to estimate model variables and parameters to satisfy input information to avoid overfitting and to correctly estimate the model uncertainty [ 56, 71] .

A whole-cell model is built by computing one or an ensemble of good-scoring models based on the modular representation determined in step 2 and the scoring function constructed in step 3. There are two alternative approaches to build the whole-cell model due to the high complexity: (i) piecewisely construct an intermediate model (or ensemble of models) for each module followed by integrating the intermediate models into a whole-cell model; (ii) globally integrate information to build a whole-cell model over modules.

Ghaemi *et al*. [19] constructed the whole-cell compartment model using the first aforementioned approach. First, intermediate kinetic models are constructed for the reaction network of RNA splicing in modules (cellular compartments) and the substrate transport among modules. The intermediate models are then integrated using common components in different compartments of the spatially-resolved cell model ( *e.g.*, uridine-rich snRNA fluxes between cytosolic and nuclear compartments) and sampled using stochastic reaction-diffusion master equations [ 19, 72] .

Similarly, the whole-cell mathematical model published by Karr *et al*. [22] is developed by constructing intermediate ODE models to represent twenty-eight modules of diverse cellular processes using various approaches ( *e.g.*, RNA and protein degradation modeled as Poisson processes and metabolism modeled using FBA [73]). The integration is then implemented by harmonizing multiple modules by common variables in different ODEs. Values of model parameters are then sampled to ensure that the intermediate models are mutually consistent by (i) resolving conflicts among the experimental data used to parameterize each module; (ii) minimizing the sum of squares deviation of model variables from experimentally observed values [22].

The whole-cell structure model integrates information over modules directly [28]. First, modules, a set of protein communities, are assembled from protein complexes to organelles. Second, neural network embeddings are computed based on immunofluorescence images in the Human Protein Atlas [54] and AP data in BioPlex [55]. Protein communities are then detected by calibrating protein distances in the embeddings into physical distances in cells using the Clique eXtracted Ontology algorithm [74]. The hierarchical protein community of the cell is obtained by sampling different parameter combinations and selecting based on two previously reported cell maps [ 75, 76] .

### Step 5: Validate and interpret the model

Once the whole-cell model is built, it should be validated by assessing the degree of consistency between the model and the information used and not used to construct the model. Two important approaches of the model validation are (i) statistical significance of the model, which is often quantified using the Student’s *t*-test and Analysis of variance; (ii) experimentally testing multiple dimensions of model predictions. In the whole-cell compartment model, for example, the ratio of localization of splicing particles in speckles is validated by the experimentally determined ratio; the time scale to generate the required abundance of splicing particles by running the whole-cell model is within the scope of human cellular lifetime, which further validates the whole-cell model [19]. The whole-cell mathematical model is validated against a broad range of independent datasets that are not used to construct the model and which describe various cell functions [22]. In the whole-cell structure model, five-fold cross validation is used when training random forest regressors for each training set. In addition, estimated diameters for all nine components are validated using actual measurements from the literature, forty-four subcellular components are validated by their enrichment for new physical interactions, identified using mass spectrometry combined with affinity purification (AP-MS) experiments [28].

Model uncertainty originates from incomplete input information, model representation incommensurate with the input information, inaccurate scoring function, and insufficient sampling [16]. In the case of sufficient sampling, the uncertainty can be estimated based on the variability in the ensemble of good-scoring models [ 23, 71] . For example, in the whole-cell compartment model, the standard deviation of the mRNA production calculated based on twenty parallel simulations reflects the model uncertainty [19]. In the whole-cell structure model, the probability density of AP-MS scores quantifies the uncertainty for the detected interactions between protein pairs for smaller, medium, large and general subcellular components [28]. Unfortunately, uncertainty quantification in biochemical models described by ODEs is still not common despite efforts to make tools for uncertainty analysis available to the field ( *e.g.*, Markov Chain Monte Carlo [77] and Bootstrap [78]) [79].

After a model is validated with proper uncertainty quantification, it is critical to interpret the model. For example, spatial or spatiotemporal models can be interpreted using various visualization tools depending on the granularity of the modeled system ( *e.g.*, ChimeraX [80], Mol* [81], PyMOL [82], CellPAINT [36], E-CELL [35] and Visual Molecular Dynamics [83]); metabolic networks in whole-cell simulations can be interpreted using WholeCellViz to provide model predictions in their biological context [84]; FBA simulations can be visualized by Escher-FBA [85]. Visualization tools await to be further developed and equipped with more interactive interfaces to allow interpreting datasets of growing size and cell models of growing variety [ 86– 88] .

### Iteratively refine the whole-cell model

When inconsistencies are found during validation and interpretation, we can iterate through the five steps of the whole-cell modeling workflow until the inconsistencies are minimized and the output model satisfies the input information within an acceptable threshold ( Figure 1). Moreover, the whole-cell model should continue to be refined iteratively with more information, more advanced modeling methods, and improved computing power. For example, many different aspects of the whole-cell mathematical model have been iteratively reconstructed with new available information which describes *Mycoplasma genitalium* physiology [22]. Specifically, the kinetics of each reaction are reconstructed based on the enzyme database BRENDA [89] and the biochemical reaction kinetics database SABIO-RK [90].

### Integrate available (whole-cell) models towards an accurate, precise and complete whole-cell model

Substantive method development to integrate existing data and models ( *e.g.*, whole-cell models) is needed to build a comprehensive whole-cell model. Bayesian metamodeling, a special case of integrative modeling, integrates models of various aspects of a cell and its parts by using any mathematical representation, scale, and level of granularity ( Figure 1) [24]. In Bayesian metamodeling, surrogate probabilistic models converted from corresponding input models of different aspects are coupled through subsets of statistically related variables (e.g, Xm and Xn in Figure 1), followed by the backpropagation to update the input models by computing the probability density functions of free parameters for each input model in the context of all other input models. The output metamodel, as any models mentioned above, is validated and interpreted before any prediction using input models, data used to construct input models, and additional available data. Bayesian metamodeling often produces a more accurate, precise, and complete description of the whole-cell that contextualizes input models as well as resolves conflicting information [24]. In particular, the integration maximizes the advantages of all models and their predictions and can reproduce the behavior of the cell not represented by any individual modules [23]. Note that methods to automate the Bayesian metamodeling workflow as well as to visualize and interpret the output metamodel await to be developed [ 23, 24] .

## Whole-cell Model Prediction on Cell Biology

In addition to providing an integration of experimental observations, a useful whole-cell model ought to be quantitatively predictive to guide biological discovery by facilitating new experimental designs [ 10, 91] . Such models allow for predicting various aspects of cell biology across the entire cells such as protein signaling and metabolic pathways to shed light on how a cell function modulates and evolves, and facilitates the discovery of drugs or therapy targets.

A list of whole-cell models and examples of their predictions are summarized in Table 1. As an example, the whole-cell mathematical model predicts not only cellular properties such as the cell mass and growth rate but also molecular properties including the number, localization, and activity of each molecule [22]. Importantly, whole-cell models provide a more complete picture of certain aspects of the entire cell, allowing us to address questions about distinct roles and relationships of functional components along with various cell signaling pathways. Several examples are new interactions between components [19], new kinetic parameters and biological functions [22], estimates of pairwise protein distances [28], and intracellular reactions that drive responses to the extracellular environment [ 92, 93] . Bayesian metamodeling, through the integration of various (whole-cell) models, facilitates model prediction by maximizing the accuracy, precision, and completeness of the whole-cell model. One example is the prediction of postprandial glucose and insulin plasma levels under the effect of glucagon-like peptide-1 (GLP-1) and incretins activating glucagon-like peptide-1 receptors (GLP-1R) for normal and type 2 diabetic subjects by integrating four heterogeneous models: a structural model of GLP-1R activation, a signaling network model of insulin secretion pathway in pancreatic β-cells, a linear model of a pancreatic cell population and a mathematical ODE model of system postprandial response. The output metamodel is expected to continue upgrading in order to provide more useful predictions to inspire more effective future experiments, discover biological mechanisms, and generate hypotheses, which will in turn enhance the whole-cell model itself [24].

## Conclusions and Perspectives

As the scales of the experimental data and the technological advancements in modeling algorithms and computing power are increasing, modeling the whole-cell will benefit from ensembling multi-scale input information by the integrative strategy. In this review, we have summarized several inspiring works that aim at modeling the whole cell from various aspects, most of which are constructed by combining disparate information over modules. Concomitantly, we propose a generally applicable integrative whole-cell modeling workflow from input information to the output whole-cell model. In particular, Bayesian metamodeling, a divide-and-conquer approach, aims to integrate (whole-cell) models of various representations into an output metamodel, potentially providing practical solutions towards mapping the entire cell. Whereas integrative strategy can in principle be applied to model the whole cell, there are still grand challenges towards modeling the whole cell that need to be conquered, including integration of vast amounts of information across multiple scales, computing capacity, visualizing and archiving existing whole-cell models and large collaborations among multiple disciplines.

Here we list a few future research directions to address the challenges. (i) Input information should grow in quantity and quality with more advanced studies and experimental communities to expand the scope of whole-cell models [ 94– 98] . (ii) New modeling methods to integrate various types of information should be developed to maximize the model accuracy, precision and sampling efficiency [23]. As an example, model representation should be optimized by objective methods and selected by formal criteria [99]. The scoring function should be ideally designed in a Bayesian fashion to improve accuracy and optimize the algorithm of quantifying the total model uncertainty of the resulting models [ 65, 100, 101] . (iii) Computing capacity needs to be improved to simulate complex whole-cell models (i.e. high-performance parallelized computing technologies [ 102– 106] ). (iv) Advances of large-data visualization are required to complement analysis technologies [98]. (v) An infrastructure to archive input models and disseminate modeling outputs helps to organize multiple types of data which is significantly exacerbated for integrative methods. Current existing asset management infrastructure ( *e.g.*, Datanator [50], Deriva [107], BioModels [59], BioStudies [61], COMBINE [108], BioSimulators [60], runBioSimulations [109] and BioSimulations [110]) can be potentially used and expanded to facilitate accessible and reproducible studies for experimentalists and modelers [11].

As the field moves forward, difficulties in experimentally detecting various cell components and in computationally simulating dynamics of and interactions among these components will be overcome. One good starting point towards a comprehensive whole-cell model is to build an integrative compartment cell model, which specifies the compartments ( *e.g.*, protein communities, microdomains and organelles) and their dynamics in a cell ( *e.g.*, location, shape and contents as a function of time) [ 39, 111] , and exchanges of molecular components among compartments ( *e.g.*, Ca ^2+^ flux among organelles [ 112– 116] ) as well as interactions between compartments [ 19, 28] ( *e.g.*, excluded volume and the boundary of the cell membrane). As an increasing number of integrative compartment cell models are established, significant roles of specific compartments, their components and interactions in various cell functions will be shed light on [ 39, 117] . This, in turn, will potentially serve as an input model for Bayesian metamodeling to compute a comprehensive and predictive whole-cell model [ 10, 24] .

Finally, large collaborations through a broadly cross-disciplinary team have been established to surmount the grand challenges of the whole-cell modeling. The Pancreatic β-Cell Consortium ( https://www.pbcconsortium.org/) aims to understand β-cell biology and diabetes by constructing spatiotemporal multi-scale whole cell models of human pancreatic β-cells; the OpenCell project in Chan Zuckerberg Biohub ( https://www.czbiohub.org/manuel-leonetti-intracellular-architecture/) aims to build a reference map of how the human cell is internally organized by combining CRISPR engineering, live-cell microscopy, and proteomics; Allen Institute for Cell Science ( https://alleninstitute.org/what-we-do/cell-science/) uses diverse technologies and approaches at a large scale to study the cell and its components as an integrated system. The whole-cell study is clearly multidisciplinary and consequently usually requires a team of collaborators, including both experimentalists and modelers [23]. Collaboration from cell biology and whole-cell modeling fields will strengthen sharing information and tools to address the knowledge gaps and measurement discreteness [118], which will accelerate the growth of quality and quantities of sophisticated whole-cell models and further contribute to designing more effective future experiments, discovering biological mechanisms of the whole cell, and facilitating drug discoveries as well as cell therapies [119].
